# Identification and Characterization of Full-Length cDNAs in Channel Catfish (*Ictalurus punctatus*) and Blue Catfish (*Ictalurus furcatus*)

**DOI:** 10.1371/journal.pone.0011546

**Published:** 2010-07-12

**Authors:** Fei Chen, Yoona Lee, Yanliang Jiang, Shaolin Wang, Eric Peatman, Jason Abernathy, Hong Liu, Shikai Liu, Huseyin Kucuktas, Caihuan Ke, Zhanjiang Liu

**Affiliations:** 1 The Fish Molecular Genetics and Biotechnology Laboratory, Aquatic Genomics Unit, Department of Fisheries and Allied Aquacultures and Program of Cell and Molecular Biosciences, Auburn University, Auburn, Alabama, United States of America; 2 State Key Laboratory of Marine Environmental Science, College of Oceanography and Environmental Science, Xiamen University, Xiamen, China; Max Planck Institute for Evolutionary Anthropology, Germany

## Abstract

**Background:**

Genome annotation projects, gene functional studies, and phylogenetic analyses for a given organism all greatly benefit from access to a validated full-length cDNA resource. While increasingly common in model species, full-length cDNA resources in aquaculture species are scarce.

**Methodology and Principal Findings:**

Through *in silico* analysis of catfish (*Ictalurus* spp.) ESTs, a total of 10,037 channel catfish and 7,382 blue catfish cDNA clones were identified as potentially encoding full-length cDNAs. Of this set, a total of 1,169 channel catfish and 933 blue catfish full-length cDNA clones were selected for re-sequencing to provide additional coverage and ensure sequence accuracy. A total of 1,745 unique gene transcripts were identified from the full-length cDNA set, including 1,064 gene transcripts from channel catfish and 681gene transcripts from blue catfish, with 416 transcripts shared between the two closely related species. Full-length sequence characteristics (ortholog conservation, UTR length, Kozak sequence, and conserved motifs) of the channel and blue catfish were examined in detail. Comparison of gene ontology composition between full-length cDNAs and all catfish ESTs revealed that the full-length cDNA set is representative of the gene diversity encoded in the catfish transcriptome.

**Conclusions:**

This study describes the first catfish full-length cDNA set constructed from several cDNA libraries. The catfish full-length cDNA sequences, and data gleaned from sequence characteristics analysis, will be a valuable resource for ongoing catfish whole-genome sequencing and future gene-based studies of function and evolution in teleost fishes.

## Introduction

A well characterized full-length cDNA set from catfish (*Ictalurus* spp.) will be crucial for studying gene duplication and gene family structures in this and closely related species, as well as aiding in the annotation of the catfish genome which is currently being sequenced. In the absence of a whole genome sequence, expressed sequence tags (ESTs) serve as important resources for gene discovery and gene identification. Reconstructing overlapping ESTs obtained by single-pass sequencing of random cDNA clones can predict transcript sequences. However, these EST reconstructions are prone to errors due to assembly of alternative splice forms, pseudogenes, and other highly similar transcript sequences including gene family members and allelic variants. The most useful transcript sequences are derived from high quality full-length cDNA sequences which contain the complete transcript in a single clone [1].

Whole genome assemblies rely on transcript sequences to stitch together contigs [2]. Full-length cDNAs, therefore, are an extremely useful tool for correct annotation and clustering of the genomic sequence in genome sequencing projects [3]. Further, full-length cDNAs are an important resource to analyze genome structure and genome function [4], [5]. Comparing full-length cDNAs to the genome build provides insight into evolution and gene regulation. Previous full-length cDNA sequencing studies have demonstrated the importance of cDNA sequences to produce gene models that establish accurate exon-intron boundaries [6], [7]. Meanwhile, full-length cDNAs are an important resource to predict protein sequences, supporting proteomic approaches [8]. Moreover, full-length cDNAs provide vital information about alternative splice forms of gene products [9] and aid in discriminating between alternative splicing and gene duplications or pseudogenes [8].

Previous studies in other agricultural species have produced full-length cDNA sets: a total of 954 bovine full-length cDNA sequences were produced to create predicted bovine protein sequences to support bovine genome assembly and functional genomic studies [8]; a database for chicken full-length cDNAs was established to provide a large amount of gene information for biological and biomedical research [10]; 560 Atlantic salmon full-length cDNAs have recently been generated for correct annotation and clustering of a forthcoming whole genome sequence [1]. Despite their usefulness, few full-length cDNAs are available in public databases for ictalurid catfish, a major aquaculture species in the United States.

Over 430,000 catfish EST sequences have been generated from the recent JGI catfish EST sequencing project [11]. The large-scale generation of EST sequences provides a platform for the identification and characterization of full-length cDNAs. In this study, we characterized and compared the full-length cDNA sequence from two closely related ictalurid catfish species, channel catfish (*Ictalurus punctatus*) and blue catfish (*Ictalurus furcatus*). Here, we report the generation and analysis of 1,767 full-length cDNAs including 1,072 from channel catfish and 695 from blue catfish. Identification of cDNA transcripts containing complete coding sequences and annotation of full-length cDNAs are presented. High similarities of open reading frame (ORF) and untranslated region (UTR) distribution were found between channel catfish and blue catfish comparing full-length cDNAs. We also examined general characteristics of catfish full-length cDNA sequences including Kozak consensus sequences, polyadenylation signal variation, and conserved motifs.

## Results and Discussion

### Selection and sequencing of clones with full-length cDNAs

As described in our previous study [11], a total of 12 cDNA libraries were constructed from various tissues, organs, and cell lines from catfish. Eight libraries for channel catfish and four libraries for blue catfish were produced. Briefly, from the previous study, 438,321 ESTs were generated and assembled into contigs and singletons [11]. From the assembly, 10,037 channel catfish and 7,382 blue catfish clones with putative full-length cDNA inserts were identified by BLASTX analysis against UniProt database with a cutoff E-value of 1e-5 (Table 1). In order to obtain high quality full-length cDNA sequences, clones were selected where the full-length cDNA sequences could be generated from single-pass sequencing and re-sequencing of individual clones, not from contig assemblies. A total of 1,169 channel catfish and 933 of blue catfish putative full-length inserts cDNA clones were selected and re-sequenced. The raw cDNA sequence base calling was conducted by using Phred [12] with a cut-off score of Q20. Manual sequence inspection was performed to ensure high quality of full-length cDNA sequences. A total of 1,793 high quality cDNA consensus sequences with a complete coding sequence were generated, including 1,087 from channel catfish and 706 from blue catfish. The average number of reads (total number of bases sequenced/total number of bases of all full-length cDNAs) of all consensus sequences was 3.76. Of total consensus cDNA sequences, 1,767 cDNAs harbored full-length cDNA sequence, and the remaining 26 were, due to minimal 3′ UTR length prior to poly (A), judged likely to contain internal poly (A) sequences. All 1,793 sequences with a complete coding sequences were submitted to Genbank (Accession numbers **GU587763—GU589555**).

**Table 1:** Summary of full-length cDNAs in channel catfish and blue catfish.

	Channel catfish	Blue catfish
Putative full-length cDNA clones	10,037	7,382
Validated full-length cDNA	1,072	695
Unique full-length genes	1,064	681
Average sequence length (bp)	1,135	1,070
Average ORF length (bp)	689	702
Average UTR length (bp)	243	250

### Length distributions of the catfish full-length cDNAs

**Figure 1:**
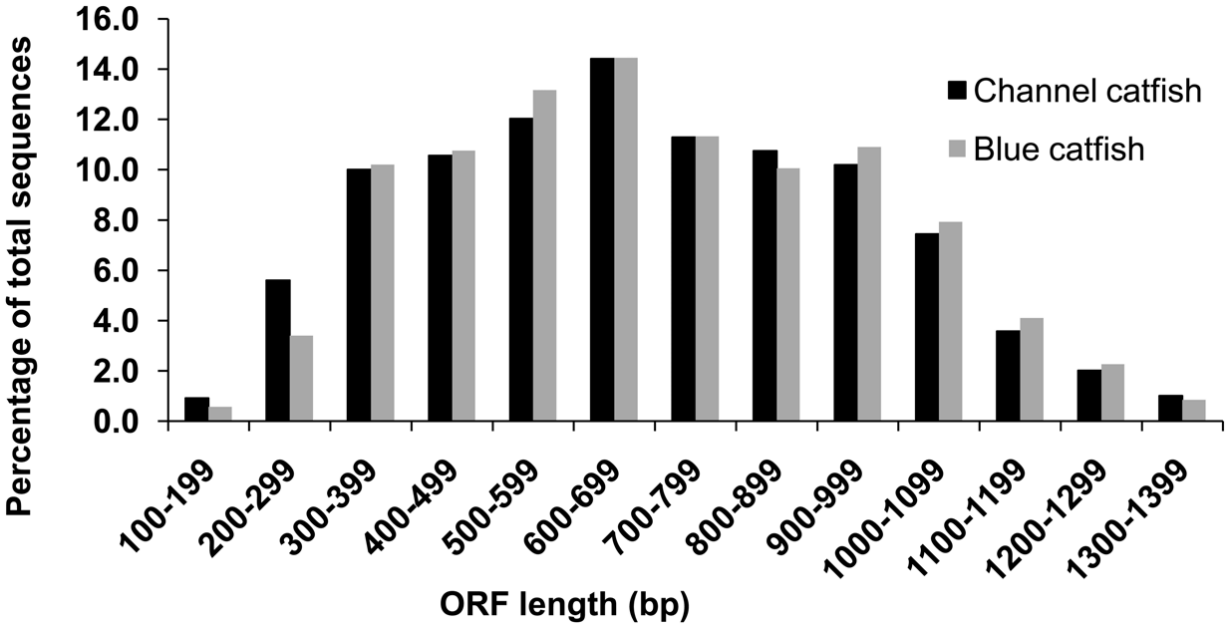
Comparison of the ORF length distribution between channel catfish and blue catfish full-length cDNAs.

**Figure 2:**
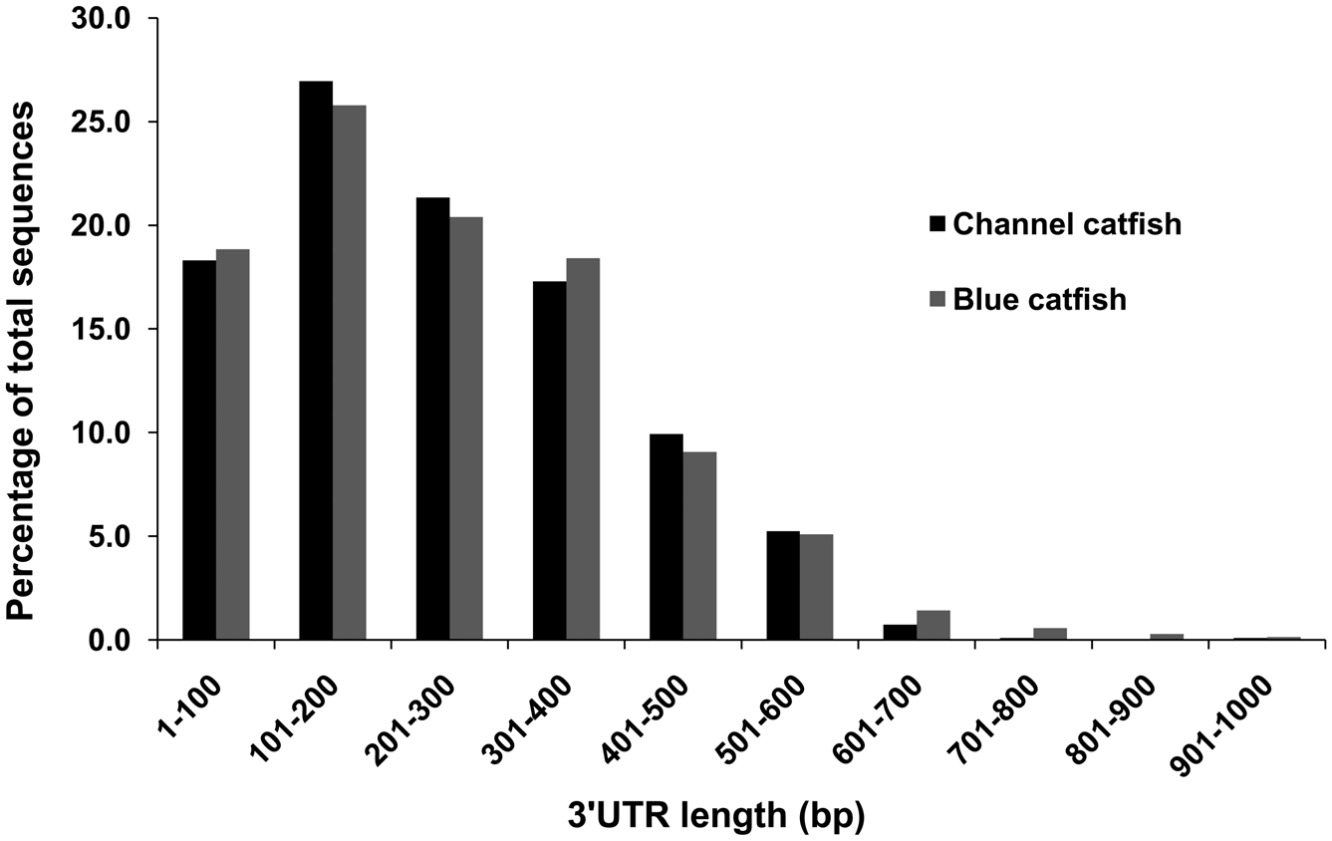
Comparison of the 3′UTR length distribution between channel catfish and blue catfish full-length cDNAs.

As shown in Table 1, the average length of the full-length cDNAs from channel catfish was 1,135 bp and 1,070 bp from blue catfish. The length of ORFs from channel catfish ranged from 114 bp to 1,416 bp, with an average ORF length of 689 bp. The range of ORF lengths from blue catfish was from 156 bp to 1,371 bp, with an average length of 702 bp. The average length of 3′UTRs from channel catfish was 243 bp, while the average length was 250 bp from blue catfish. Channel catfish and blue catfish full-length cDNAs exhibited similar patterns of distribution of ORF and 3′UTR length (Figures 1 and 2). The majority of ORF lengths ranged from 300 bp to 999 bp, while most of the 3′UTRs had lengths less than 400 bp. The analysis through the identification of ORFs and UTRs had the strength of detecting protein coding capacity and provided valuable information allowing comparisons of the distribution pattern between the two ictalurid catfish species. However, given the bias of cDNA library creation and our selection process towards smaller transcripts, the results likely do not represent comprehensive ORF and UTR distributions in the catfish transcriptome.

### Gene identification, gene ontology and mapping

A total of 1,745 unique gene transcripts were identified by BLASTX, including 1,064 gene transcripts from channel catfish and 681 gene transcripts from blue catfish, by searching against the UniProt protein database (Table 1). Of the 1,745 catfish gene transcripts, 416 transcripts shared the same gene identity between blue catfish and channel catfish.

**Figure 3:**
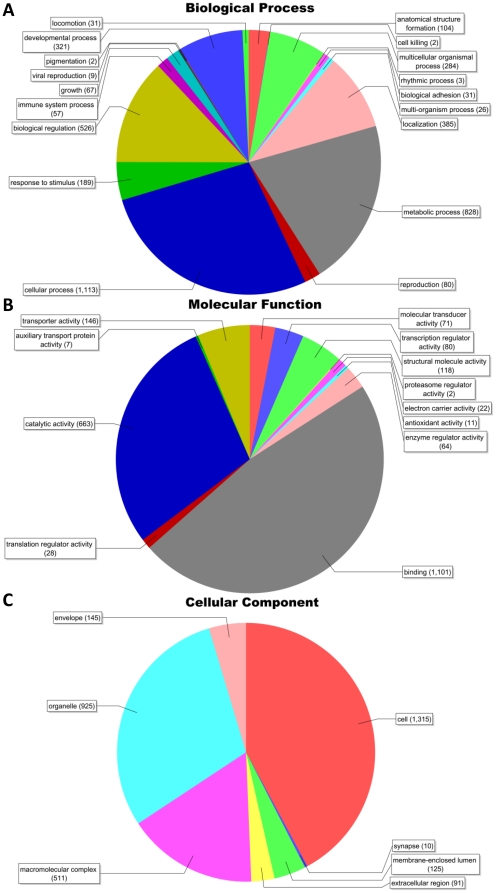
Distribution of most common GO terms in the categories molecular function. A, Biological Process; B, Molecular Function; C, Cellular Component.

Gene ontology (GO) [13] searches were performed using all complete coding cDNA sequences (1,793) using Blast2GO annotation software [14] with E-value <1e-10. At 2^nd^ level GO, 1,268 sequences were assigned to the biological process category (Figure 3A), 1,381 sequences to the molecular function category (Figure 3B), and 1,348 sequences to the cellular component category (Figure 3C). From the GO category of biological process, cellular process was the most dominant term, accounting for 87.8% of sequences annotated in that category, followed by metabolic process (65.3%). In the molecular function category, binding (79.7% of sequences) was the most dominant term, followed by catalytic activity (48%). A similar distribution pattern was found when all catfish ESTs were subjected to same gene ontology searches [11]. Cellular process (74%) was the most dominant term from the GO category of biological process, followed by metabolic process (58%). In the molecular function category of all catfish EST GO analysis, binding (61%) was the most dominant, followed by catalytic activity (51%) [11]. Comparison of gene ontology composition between generated full-length cDNAs and all catfish ESTs revealed that the full-length cDNA set was representative of the gene diversity encoded in the catfish transcriptome.

Physical and genetic linkage maps are considered important tools for position-based cloning of economically important genes and genome studies. In order to locate full-length cDNAs on the catfish BAC-based physical map and genetic linkage map, all full-length cDNAs were searched against catfish BAC end sequences (BES) [15] with significance of E-value < 1e-20. A total of 147 gene hits were identified, including 86 from channel catfish and 61 from blue catfish. All 147 gene hits against BES were located in 101 BAC contigs on the catfish physical map. Through ongoing integration of the catfish physical and linkage maps, forty-one of the full-length cDNAs could be placed on the catfish genetic linkage map, distributed among nineteen linkage groups (Table S1).

### Comparison of full-length cDNA sequences between channel catfish and blue catfish

**Figure 4:**
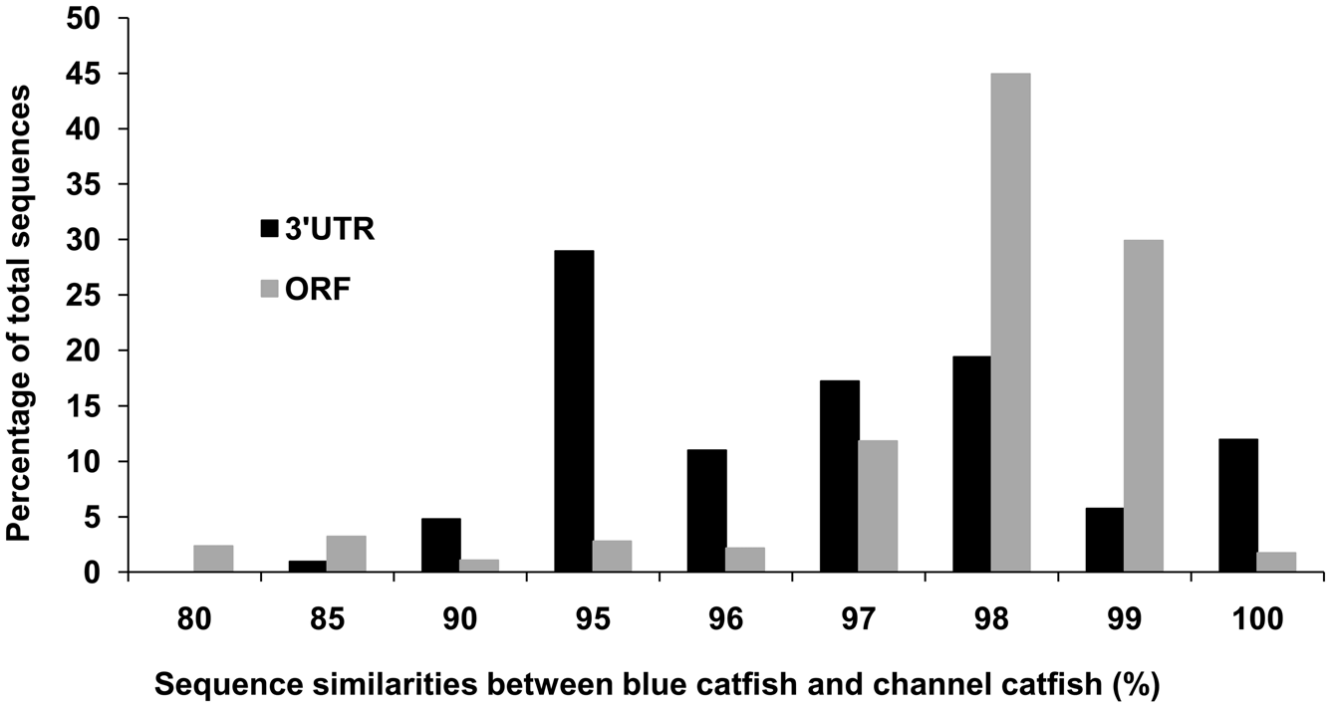
Comparison of ORF and 3′UTR length. Comparison of ORF and 3′UTR length between channel catfish full-length transcripts and blue catfish full-length transcripts with the same BLAST identities. Similarities were rounded to the nearest whole number.

Of all catfish full-length cDNA sequences, 416 genes with matching UniProt BLASTX identities were sequenced in both channel catfish and blue catfish with high similarities, ranging from 80% to 100%. As shown in Figure 4, a large majority of the ORFs shared between the two species had 98% to 99% similarities (74.8% of total sequence). The similarity of UTRs from the shared sequences was also high, albeit lower than that seen in the ORF region. The largest fraction of the shared UTRs had 95% or greater similarity between the two species.

### Kozak motif and polyadenylation signal (PAS) in catfish transcripts

**Figure 5:**
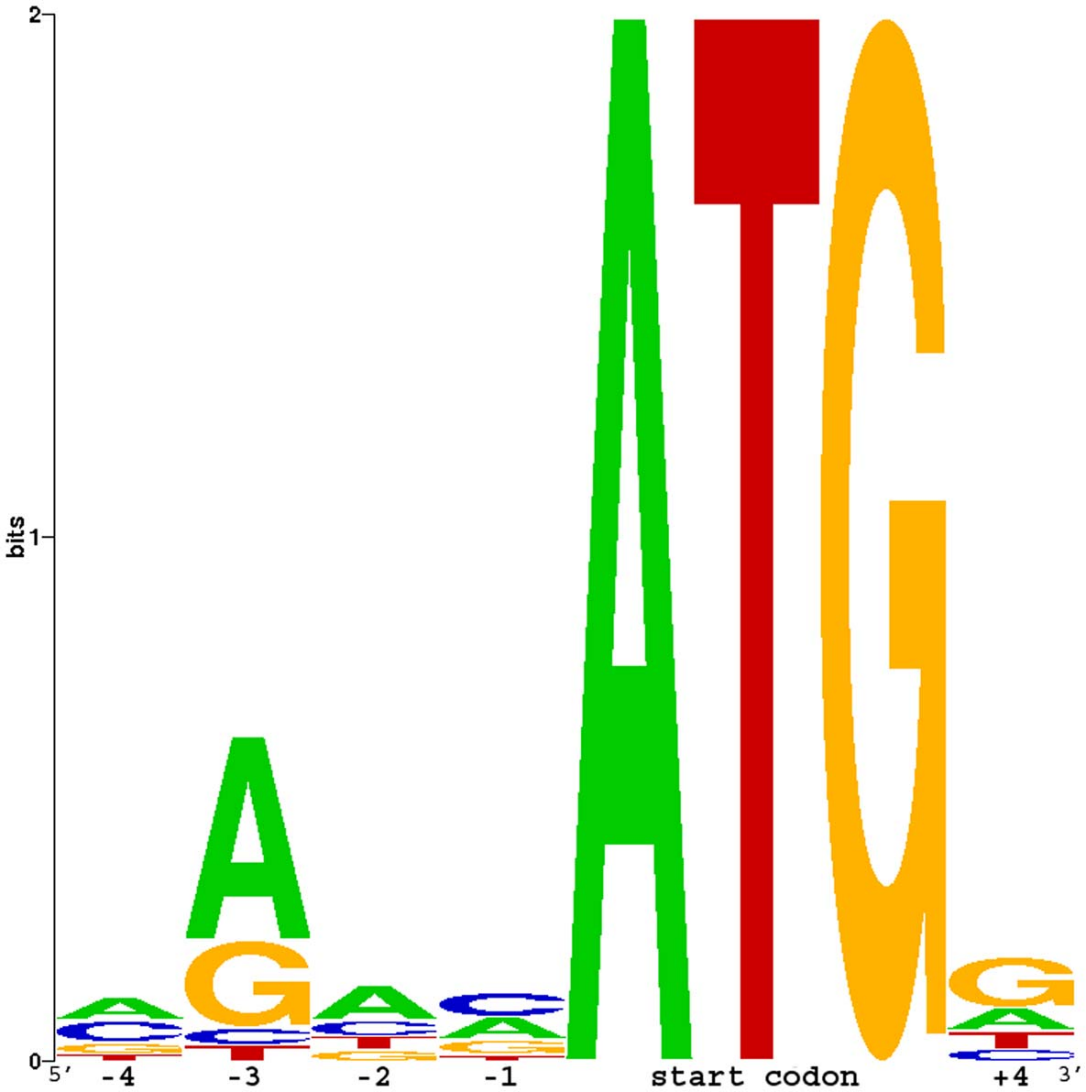
Kozak consensus sequences in catfish. Kozak consensus sequences surrounding start methionine in catfish full-length cDNAs were illustrated by WebLog.

The AUG start codon context, referred to as the Kozak motif, was reported as a consensus sequence for initiation of translation in vertebrates [16]. WebLogo [17] was used to illustrate the catfish Kozak motif, spanning the position −4 to +4. As shown in Figure 5, the most frequently observed bases in catfish Kozak motifs based on extracted 8 bp sequences were AAACATGG, with the start codon underlined. Although some of the 5′UTRs were short and incomplete, perhaps due to library construction, a large majority of the full-length cDNAs had enough sequences immediately upstream of the start codon to allow Kozak sequence motif analysis. The most highly conserved base in the Kozak motif was a purine in the -3 position [16]. In mammals, the consensus Kozak motif sequence is reported as CACCATGG, with the start codon underlined [8]. The most frequently observed bases in the Kozak motif of Atlantic salmon, *Salmo salar*, were CAACATGG [1]. Catfish Kozak sequences appear to be highly similar to *S. salar* except an adenine base instead of cytosine base was found at the -4 position.

The polyadenylation signal (PAS) is an important component of the transcription process where a stretch of adenines determines polyadenylation. The mature 3′UTR is formed by polyadenylation of the pre-mRNA, a coupled reaction affecting mRNA stability and translation. The most essential sequence component required for polyadenylation is a highly conserved PAS. Several studies reported that different variants of the PAS exist, and that the frequency distribution of the most common PAS was species-dependent [18], [19]. Different variants of the PAS were observed in catfish transcripts from this study. The most common PAS observed immediately upstream of the poly (A) tail (within 35 bp) was the canonical AAUAAA (973 transcripts, 55%). The second most common variant was AUUAAA, present in 467 transcripts and accounting for 26%. To reveal the most frequently occurring hexamers in the remaining transcripts, the TEIRESIAS algorithm [20] was used. The result showed that no dominant hexamer accounted for the remainder of the transcripts. Rather, several additional hexamers, including some identified as putative polyadenlyation signals in salmon, were identified. Table 2 shows the ten most frequent hexamers, present in 30 or more transcripts. Of those ten putative PASs, UAUAAA, AUAAAA and AAUAUA have been reported as PAS variants in salmon transcripts [1].

**Table 2:** Most common hexamers identified in 3′UTRs from catfish transcripts.

Sequences of hexamer	Number of transcripts
UAUAAA*	48
AAAAAA	43
AUAAAA*	40
UGUUUU	38
UAAAAA	38
AAAAAC	34
UUUUUG	33
UUUAAA	33
AGUAAA	32
AAUAUA*	30

*The hexamers were reported as putative polyadenylation signals in salmon transcripts [1].

In gene regulation, which motif is present as the PAS may be important. By far, the most frequently found PASs were AAUAAA and AUUAAA. It has been reported that in human transcripts, AAUAAA and AUUAAA are present in 58.2% and 14.9% of the 3′UTRs, respectively [18]. Similar ratios were observed in our result; in catfish, AAUAAA and AUUAAA were observed in 54.3% and 26% of the 3′UTRs respectively.

### Conserved candidate regulatory motifs in catfish transcripts

The UTRs are usually rich in motifs which regulate gene expression by binding various trans-acting factors. In this study, the UTR analysis of full-length cDNAs provided an opportunity to identify putative functional regulatory motifs in catfish transcripts. All 5′ and 3′UTRs from catfish transcripts were searched against the UTR database collection (UTRdb) [21] by using the pattern matcher program UTRscan. The analysis of the 5′UTRs revealed a transcript annotated as ferritin with a motif that matched the iron responsive element (IRE) (Table 3). IRE, a particular hairpin structure, is recognized by trans-acting proteins known as iron regulatory proteins [22]. This evolutionary conserved motif was known to be present in the ferritin gene of vertebrates [23]. Our observation that an IRE was located in the 5′UTR of catfish ferritin supported the idea that the motif identified was a true functional IRE. The analysis of the 3′UTRs of the full-length cDNAs also revealed seven transcripts with motifs that matched Selenocysteine Insertion Sequences (SECIS). The SECIS element is a specific 60 bp stem-loop structure located in 3′UTRs of mRNAs, and required for decoding UGA selenocysteine instead of termination of translation [24]. Catfish transcripts with matches to the SECIS element encoded selenium-related genes, such as glutathione peroxidase [25], defender against cell death protein [26], selenoprotein and Glutaredoxin [27]. Additionally, five catfish transcripts encoding dynein, cysteine-rich protein and transmembrane protein contained 3′UTR motifs that matched the alcohol dehydrogenase 3′UTR downregulation control element (ADH_DRE; Table 3).

**Table 3:** Conserved candidate regulatory motifs in UTRs from catfish transcripts.

GeneBank ID	Annotation	Regulatory Motifs
GU588191	Ferritin	IRE
GU588163	Glutathione peroxidase 4a	SECIS
GU588186	Defender against cell death	SECIS
GU588757	Glutaredoxin-1	SECIS
GU588767	Selenoprotein X	SECIS
GU588938	Glutathione peroxidase 4b	SECIS
GU589011	Glutathione peroxidase 1b	SECIS
GU589059	Defender against cell death	SECIS
GU587798	Dynein	ADH_DRE
GU588188	Cysteine-rich protein 1	ADH_DRE
GU588727	Transmembrane protein	ADH_DRE
GU589032	Cysteine-rich protein 1	ADH_DRE
GU589100	Dynein	ADH_DRE

IRE: Iron Responsive Element; SECIS: Selenocysteine Insertion Sequences; ADH_DRE: Alcohol Dehydrogenase 3′UTR Downregulation control Element.

### Identification of paralogous sequences

**Figure 6:**
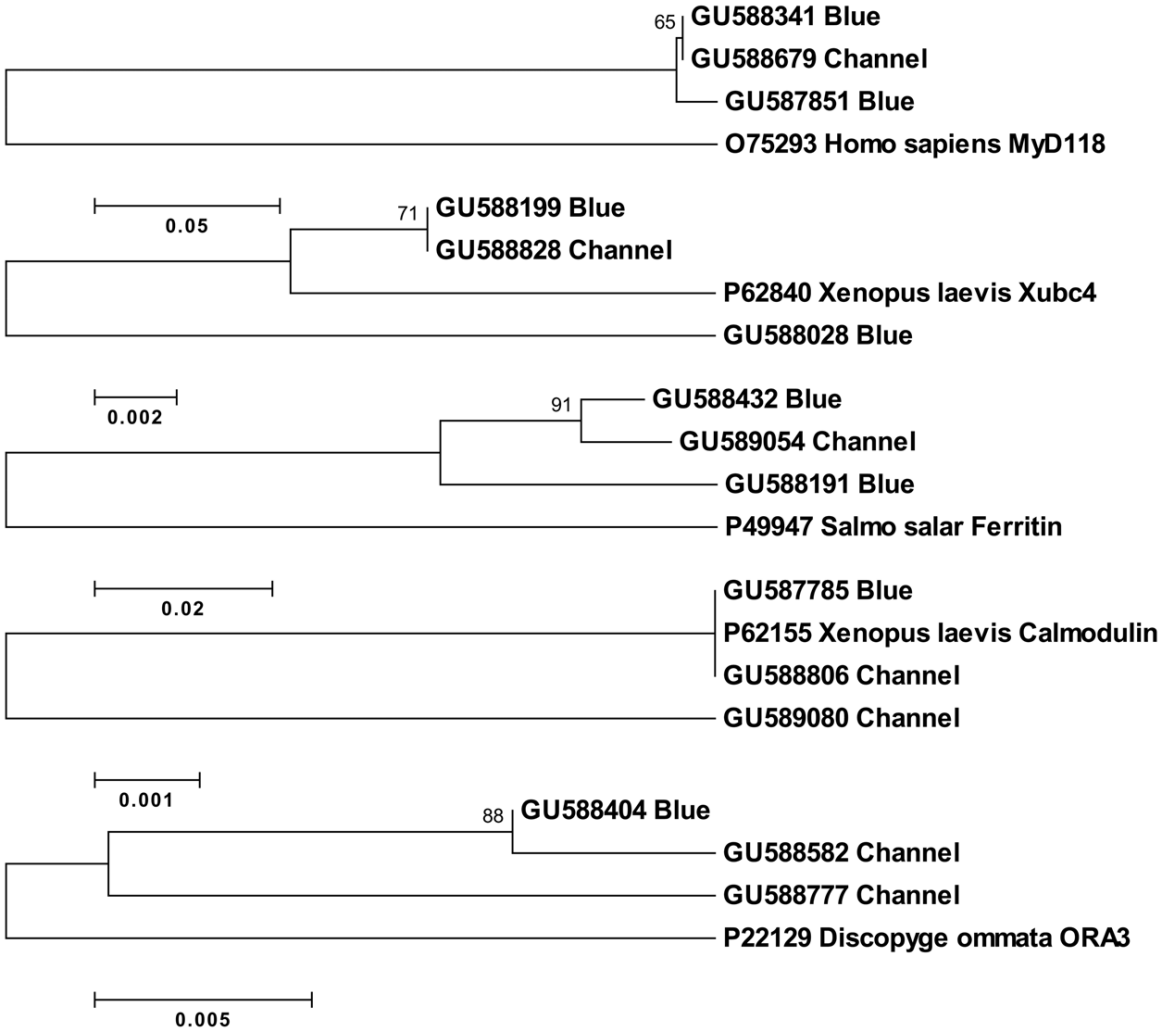
Phylogenetic analysis of putative catfish paralogues. Highly similar channel catfish sequences (Channel) and one blue catfish sequence (Blue) sharing the same BLAST identity, or two highly similar blue catfish sequences with one channel catfish sequence sharing the same BLAST identity, were subjected to phylogenetic analysis. The phylogenetic tree was drawn by using the neighbor-joining method in MEGA 4.0 package. The topological stability of the tree was evaluated by 1,000 bootstrap replications. Data were analyzed using Poisson correction and gaps were removed by complete deletion.

In the absence of a catfish whole genome sequence, full-length cDNAs may provide valuable information in the analysis of gene duplication events. Consensus sequences are often constructed from partial fragments of allelic variants and/or paralogous sequences, complicating analyses of gene copy numbers and gene families. Based on sequence similarity, shared UniProt BLASTX identity and GO annotation, there were several catfish full-length transcripts, with high sequence identity, from 79% to 99%, likely representing paralogues. However, it was difficult to differentiate between gene duplication and allelic variation if only comparing highly similar transcripts from a given species. Using a two species (channel catfish and blue catfish) approach with full-length cDNA sequences may help to distinguish gene duplication from allelic variants [28]. Using the rationale that allelic variation within the same species should be smaller than the variation present between orthologues from different species [29], two potentially paralogous sequences from either channel catfish or blue catfish and a putatively orthologous sequence with the same UniProt BLASTX identity from the other species (channel or blue) were subjected to phylogenetic analysis. When channel catfish and blue catfish orthologues were more tightly grouped on the resulting tree than the additional, highly similar sequence from blue or channel catfish, the two sequences from the same species likely represented paralogues rather than allelic variants. Assuming that the evolutionary distance of allelic variants should be closer than that of orthologues from a closely related species, there were two channel catfish paralogs and three blue catfish paralogs identified from the full-length cDNA set (Figure 6). Additional expansion of full-length cDNA sets from both blue catfish and channel catfish in the future should allow validation and extension of this approach to gene duplication analysis.

In conclusion, full-length cDNA sequences are important resources for functional and structural genome studies. This paper described the first catfish full-length cDNA set constructed from several cDNA libraries. In total, 1,767 high quality catfish full-length cDNAs were generated and analyzed. With catfish whole genome sequencing in progress, full-length cDNAs will be a valuable resource for researchers, allowing correction of assemblies, annotation, and construction of gene models that establish accurate exon-intron boundaries.

## Materials and Methods

### Identification of putative full-length cDNAs

All JGI catfish ESTs [11] were utilized for identification of putative full-length cDNAs. In this previous effort [11], cDNA clones were sequenced from both 5′and 3′ends to generate 438,321 ESTs; assembly was conducted on this set of ESTs using the Paracel Transcript Assembler (PTA; based on the CAP3 program) [30]. All unique sequences obtained from the assembly were analyzed using TargetIdentifier [31] to identify assembled sequences that were generated from clones harboring putative full-length cDNA inserts. The program utilized both UniProt BLASTX results and start/stop codon usage. Where a catfish sequence had a significant (E<e-5) BLASTX hit against a UniProt sequence and contained an in-frame ATG further upstream (5′), at the same position, or within the first 10 amino acids of the reference sequence, the catfish cDNA sequence was considered to encode a conserved start codon.

### Re-sequencing of cDNA clones with full-length cDNA inserts

In order to avoid the creation of pseudo-haplotypes, a single representative cDNA clone from each putative full-length cDNA was selected for re-sequencing. Only clones that had an overlapping 5′ and 3′ EST sequences when assembled were selected. All selected cDNA clones were picked from −80°C stocks, transferred to 96-well culture blocks containing 1.5 ml of LB broth and the appropriate antibiotic (50 µg/ml chloramphenicol or ampicilin), and cultured overnight at 37°C. Plasmid isolation was performed using the Perfectprep® Plasmid 96 Vac Direct Bind Kit (Eppendorf, Hauppauge, NY). Clones were sequenced from both the 5′- and 3′-end using Big Dye v.3.1 terminator (Applied Biosystems, Foster City, CA) and universal primers based on the library, including M13 F (5′TGTAAAACGACGGCCAGT3′), M13 R (5′CAGGAAACAGCTATGACC3′), T7 (5′TAATACGACTCACTATAGGG3′) and SP6 (5′ATTTAGGTGACACTATAG3′). Sequencing was performed on an ABI 3130*xl* Genetic Analyzer (Applied Biosystems) following manufacturer's protocol.

### Sequence analysis, gene identification and gene ontology

VectorNTI version 10 (Invitrogen, Carlsbad, CA) was used for sequence assembly. Raw ABI sequence trace files from selected clones in this study as well as all trace files from the JGI were assembled into contigs using ContigExpress, with criteria of 80% identity and 40 bp overlap. Each assembly contained four sequences: 5′- and 3′-end sequences from both JGI sequencing data and our re-sequencing effort. Each contig sequence was manually screened for sequencing errors using chromatograms and quality scores to assess any sequence variation, where applicable. Contig sequences were further manually screened for full-length sequences using BLASTX alignments, by searching against the *nr* database. Raw cDNA sequence base calling and trimming was conducted by using Phred with a cut-off score of Q20. The average quality score for the full-length cDNA set was Q50. In this study, a full-length cDNA is defined as a sequence derived from a single cDNA clone with a conserved start codon, a complete ORF, a stop codon and a poly (A) tail.

To obtain putative gene identities, BLASTX against the UniProt databases with a cutoff E-value of 1e-10 was used. BLASTX results were used to obtain gene ontology annotation using the Blast2GO program. The annotation was conducted under default Blast2GO parameters. GO ontology can also be viewed in Table S1.

### Location of full-length cDNAs on catfish physical map and genetic linkage map

To locate the full-length cDNAs on our previous BAC-based physical map and microsatellite-based genetic linkage map, all full-length cDNAs were searched against catfish BESs by conducting BLASTN with significance of E-value < 1e-20. Only BES hits with the same UniProt BLASTX gene identity as the matching full-length cDNA were selected. Based on the location of identified BES hits on the catfish BAC-based physical map, the full-length cDNAs were located on the physical map. Positions of the full-length cDNAs on the catfish genetic linkage map were identified by anchoring microsatellite markers derived from the contig where the full-length cDNA was located (Table S1).

### Analysis of UTRs of full-length transcripts

For 5′ UTR analysis, the catfish Kozak consensus sequence was examined. Eight bp sequences spanning from position -4 to position +4 of transcripts were selected. These sequences were extracted and aligned using ClustalW. The alignment file was used for input into WebLogo [17] to assess the common Kozak consensus sequence in catfish.

For 3′UTR analysis, a web-based program was used to search putative polyadenylation signals (PAS) in full-length cDNAs. First, poly (A) tails were identified and removed from sequences. The next 35 bp of the sequence immediately upstream of the poly (A) tail were selected and manually screened for the most common PAS (AAUAAA and AUUAAA). The remaining transcripts without one of the two PAS were screened for common hexamers using the TEIRESIAS-based pattern discovery tool. Pattern discovery tool conditions in the program were set to “exact discovery”, L = 6, W = 6.

To search conserved candidate regulatory motifs in catfish transcripts, all 5′ and 3′ UTRs were used as queries to search against the UTRdb by using pattern matcher program UTRscan.

### Phylogenetic analysis

All channel catfish and blue catfish full-length cDNA sequences were used to conduct BLASTX searches against the UniProt database. Sequences that shared the same top BLASTX hit were selected for phylogenetic analysis. Phylogenetic trees were constructed using the neighbor-joining method within the Molecular Evolutionary Genetics Analysis (MEGA 4.0) package [32]. The topological stability of the trees was evaluated by 1,000 bootstrap replications. Settings included Poisson correction and gaps removed by complete deletion.

## Supporting Information

Table S1 Full-length cDNA information including GenBank accession number, BLASTX Gene_ID, BAC end sequence (BES) ID, physical map contig ID, linkage group ID, top gene hit name, and gene ontology (GO) annotation
(0.57 MB XLS)
